# Modeling Brain Volume Using Deep Learning-Based Physical Activity Features in Patients With Dementia

**DOI:** 10.3389/fninf.2022.795171

**Published:** 2022-03-09

**Authors:** Bumhee Park, Byung Jin Choi, Heirim Lee, Jong-Hwan Jang, Hyun Woong Roh, Eun Young Kim, Chang Hyung Hong, Sang Joon Son, Dukyong Yoon

**Affiliations:** ^1^Department of Biomedical Informatics, Ajou University School of Medicine, Suwon-si, South Korea; ^2^Office of Biostatistics, Ajou Research Institute for Innovative Medicine, Ajou University Medical Center, Suwon-si, South Korea; ^3^Department of Biomedical Systems Informatics, Yonsei University College of Medicine, Yongin-si, South Korea; ^4^Department of Psychiatry, Ajou University School of Medicine, Suwon-si, South Korea; ^5^Department of Brain Science, Ajou University School of Medicine, Suwon-si, South Korea; ^6^Department of Biomedical Sciences, Ajou University Graduate School of Medicine, Suwon-si, South Korea; ^7^Center for Digital Health, Yongin Severance Hospital, Yonsei University Health System, Yongin-si, South Korea; ^8^BUD.on Inc., Jeonju-si, South Korea

**Keywords:** dementia, cognitive dysfunction, accelerometer, actigraphy, autoencoder, deep learning

## Abstract

There is a proven correlation between the severity of dementia and reduced brain volumes. Several studies have attempted to use activity data to estimate brain volume as a means of detecting reduction early; however, raw activity data are not directly interpretable and are unstructured, making them challenging to utilize. Furthermore, in the previous research, brain volume estimates were limited to total brain volume and the investigators were unable to detect reductions in specific regions of the brain that are typically used to characterize disease progression. We aimed to evaluate volume prediction of 116 brain regions through activity data obtained combining time-frequency domain- and unsupervised deep learning-based feature extraction methods. We developed a feature extraction model based on unsupervised deep learning using activity data from the National Health and Nutrition Examination Survey (NHANES) dataset (*n* = 14,482). Then, we applied the model and the time-frequency domain feature extraction method to the activity data of the Biobank Innovations for chronic Cerebrovascular disease With ALZheimer’s disease Study (BICWALZS) datasets (*n* = 177) to extract activity features. Brain volumes were calculated from the brain magnetic resonance imaging of the BICWALZS dataset and anatomically subdivided into 116 regions. Finally, we fitted linear regression models to estimate each regional volume of the 116 brain areas based on the extracted activity features. Regression models were statistically significant for each region, with an average correlation coefficient of 0.990 ± 0.006. In all brain regions, the correlation was > 0.964. Particularly, regions of the temporal lobe that exhibit characteristic atrophy in the early stages of Alzheimer’s disease showed the highest correlation (0.995). Through a combined deep learning-time-frequency domain feature extraction method, we could extract activity features based solely on the activity dataset, without including clinical variables. The findings of this study indicate the possibility of using activity data for the detection of neurological disorders such as Alzheimer’s disease.

## Introduction

The number of patients with dementia and the associated social burdens are rapidly increasing due to global aging (Rizzi et al., 2014). To date, there has been no effective treatment for recovering decreased cognitive function once dementia has progressed. Therefore, it is essential to detect dementia as early as soon as possible before the condition worsens and to continuously monitor the symptoms of early dementia for timely and proper intervention (Robinson et al., 2015). It is well known that cognitive decline in dementia is associated with brain volume reductions across broad regions (Jack et al., 2005; Misra et al., 2009). Patients with pre-dementia [i.e., those with mild cognitive impairment (MCI)] experience brain atrophy even in the early stage of the disease, and such patients are more prone to progress to dementia (Jack et al., 2005; Misra et al., 2009).

Currently, magnetic resonance imaging (MRI) is the most reliable tool for identifying brain volume; however, an MRI examination inevitably requires a deliberate decision to schedule a costly and time-consuming visit to a hospital that is equipped with MRI machines, followed by additional follow-up scheduling. These limitations make early diagnosis of dementia and the follow-up in patients in the early stages difficult. Thus, recent studies have suggested the use of feasible alternative data for estimating brain volume and for predicting prognoses, supplementing the limitations of MRI-based examinations. For one such application, studies have shown that patient physical activity data collected from accelerometers are highly related to brain volumes (Klaren et al., 2015; Arnardottir et al., 2016; Tan et al., 2017; Spartano et al., 2019). In addition, using accelerometers offers several advantages; (i) accelerometer sensors objectively measure real-world activities of the subjects, (ii) their use is much less costly than MRI, and (iii) they can be used for continuous measurements. The rate of adoption of wearable devices is also anticipated to increase steadily in the future. However, an analytical limitation remains, despite previous efforts showing associations between accelerometer data and brain volume. Since activity data are typically unstructured and not easily interpretable, classical statistical approaches might allow us to use only intuitive, partial information in the form of original, raw physical activity data. For instance, previous studies have used the daily step count (Alosco et al., 2015; Arnardottir et al., 2016) or the time spent performing light physical activity, sedentary activity, or moderate–vigorous physical activity (MVPA) (Klaren et al., 2015; Spartano et al., 2019). Vast, non-linear attributes of activity data may remain obscure; characterizing associations between information and new features of dementia could further improve the estimation of brain volume.

An autoencoder model can capture such invisible and non-linear information effectively. An autoencoder is an unsupervised deep learning model composed of an encoder and a decoder (Kramer, 1991; Jang et al., 2020). An encoder receives high-dimensional input data and encodes it into a low-dimensional latent vector. The decoder receives a latent vector and outputs that data to facilitate the reconstruction of the input data as much as possible. Thus, only essential information is compressed in the latent vector during the learning process of the autoencoder. Eventually, the encoder component of an autoencoder could be used as a feature extraction model; such models have demonstrated reliable degree of performance in settings involving atypical and large-scale data (Hinton and Salakhutdinov, 2006).

Additionally, previous studies have associated physical activity data only with the total brain volume or total gray matter content; they have not shown detailed associations with each regional volume. However, various neurological diseases, including dementia, exhibit differential abnormalities in regional volumes in multiple areas. For example, even in the early stage, Alzheimer’s disease (AD) is accompanied by atrophy in various limbic and cingulate regions (Saykin et al., 2006; Johnson et al., 2012). Furthermore, it is known that atrophy of these regions in MCI patients is a prognostic factor for progression to AD within a few years (Hämäläinen et al., 2007; Fan et al., 2008).

Thus, this study aimed to model local brain volume in dementia patients through the analysis of meaningful physical activity features estimated by an autoencoder model and combined with features obtained with a time-frequency domain extraction method. To accomplish this, we extracted the appropriate features from physical activity data and considered their correlation with brain volumes. Accordingly, we subdivided the entire brain into 116 distinct regions (90 cerebral regions and 26 cerebellar regions) defined by automated anatomical labeling (AAL) delineation (Desikan et al., 2006). We measured each regional volume using a voxel-based morphometry (VBM) – Diffeomorphic Anatomical Registration Through Exponentiated Lie Algebra (DARTEL) procedure (Friston, 1994; Wright et al., 1995; Penny et al., 2011). Subsequently, we regressed each regional volume onto the physical activity features provided by the autoencoder model and time-frequency domain feature extraction.

## Materials and Methods

### Recruitment

This study was approved by the Ajou University Hospital Institutional Review Board (AJIRB-MED-EXP-17-470). Before collecting accelerometer data (i.e., physical activity data) from patients at Ajou University Hospital, we received their informed consent (Biobank Innovations for chronic Cerebrovascular disease With ALZheimer’s disease Study, BICWALZS). The other dataset used in this study, the National Health and Nutrition Examination Survey (NHANES), is publicly available (Centers for Disease Control and Prevention [CDC], 2003, 2005). All data were de-identified and used only for this retrospective study.

### Study Overview

**FIGURE 1:**
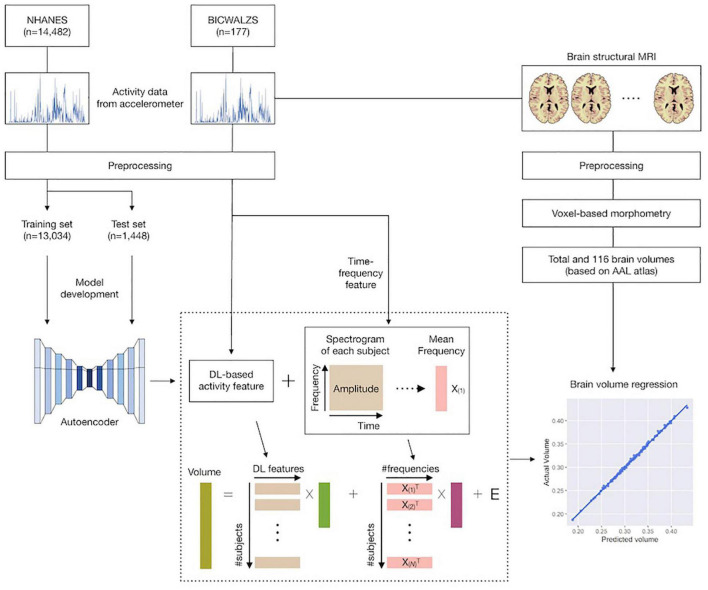
Schematic overview of the study. Accelerometer data from the NHANES and BICWALZS were collected and preprocessed. Using an unsupervised deep learning approach, the feature extraction model was developed using the activity data in the NHANES dataset; subsequently, activity features were extracted from the BICWALZS activity dataset using the developed feature extraction model together with the time-frequency domain feature extraction model. Finally, linear regression models that estimated the volumes of 116 regions based on the extracted activity features were constructed. NHANES, National Health and Nutrition Examination Survey dataset; BICWALZS, Biobank Innovations for chronic Cerebrovascular disease With ALZheimer’s disease Study; MRI, magnetic resonance imaging; AAL, automated anatomical labeling.

This study consisted of the following five stages: (1) we collected and preprocessed accelerometer data from the NHANES and BICWALZS; (2) a feature extraction model was developed based on an unsupervised deep learning approach (i.e., an autoencoder model) using the activity data from the NHANES dataset; (3) using both the previously developed feature extraction model and the traditional time-frequency domain feature extraction method, activity features were extracted from the BICWALZS activity dataset; (4) individual brain volumes were calculated from brain MRI assessments of patients enrolled in the BICWALZS using the VBM-DARTEL procedure (Ashburner and Friston, 2000), and the brain was subdivided into 116 regions based on an AAL map (Desikan et al., 2006; Spartano et al., 2019) to discover the relationship between the extracted activity features and brain volumes, we constructed linear regression models that estimated the total brain volume and the volumes of the 116 regions based on the extracted activity features. More details of the study design are described in Figure 1.

### Data Sources

#### National Health and Nutrition Examination Survey Dataset

The NHANES dataset includes accelerometer data, which were collected over 4 years (2003–2006) from 14,482 individuals living in the United States. The NHANES participants were advised to wear a physical activity monitor for seven consecutive days and to remove the device at bedtime. The device used in the NHANES was the AM-7164, manufactured by ActiGraph (John and Freedson, 2012). The device is programmed to detect and record the intensity of acceleration, which was each summed over 1-min epochs and stored in memory for every 1-min time interval.

#### Biobank Innovations for Chronic Cerebrovascular Disease With ALZheimer’s Disease Study Dataset

The BICWALZS has collected diverse kinds of data (e.g., brain MRI, activity data) from patients who visited a tertiary hospital with MCI, subjective memory impairment, AD, or all other varieties of dementia. Patients who had a brain lesion with neuropathy, seizure, alcoholic dementia, or other psychiatric disorders were excluded from the recruitment. Included in the BICWALZS dataset were physical activity and brain MRI data, as well as demographic information for 177 patients, collected in 2014–2018 at Ajou University Hospital in South Korea. Patients provided written informed consent before data collection.

At the first medical examination, brain MRI was conducted; immediately afterward, patients received an accelerometer and were instructed to wear it, even during sleep. The accelerometer used in the study was the Fitmeter, manufactured by FitNLife (Kim et al., 2014). Due to battery problems associated with the device, participants were required to visit the hospital every 2 weeks to replace the device. The average length of time the device was worn by each subject in the study was 24.6 days. Detailed information about the accelerometers and the MRI parameters for the NHANES and BICWALZS datasets are provided in Supplementary Table 1.

### Data Processing

#### Accelerometer

We preprocessed two activity datasets according to the following procedure. First, the data formats of the two databases were transformed into the same format. Because the NHANES activity data were collected in 1-week lengths, at 1-min intervals, and as 1-axis acceleration values, only the first 1-week dataset of the BICWALZS was utilized, and the triaxial accelerometer data were aggregated into a single vector by applying a vector magnitude formula and summing over each 1-min interval. Subsequently, the activity data from the two data sources were normalized via standard normalization.

As a result, each record from the NHANES and BICWALZS consisted of 1,440 values per day, with each value representing the amount-of-activity value over 1 min. Next, we eliminated the activity data collected during the nighttime (9:00 p.m. to 9:00 a.m.) because the participants in the NHANES were recommended not to wear the accelerometer at bedtime (Centers for Disease Control and Prevention [CDC], 2005). Finally, we excluded the days for which missing values were present over more than 30 consecutive minutes.

#### Preprocessing of Magnetic Resonance Imaging Data

To extract the regional gray matter volumes (rGMV), VBM analysis was performed using the VBM-DARTEL procedure in Statistical Parametric Mapping (SPM) software, version 12 (SPM12^1^, Wellcome Trust Centre for Neuroimaging, London, United Kingdom) (Friston, 1994; Wright et al., 1995; Penny et al., 2011). No abnormalities due to motion and/or other artifacts were found on the T1-weighted images following inspection by a well-trained physician. T1-weighted image preprocessing included the following: (i) gray matter segmentation based on a standard tissue probability map provided by SPM, (ii) creation of a study-specific template, spatial normalization using DARTEL to normalize individual images to the DARTEL template, and modulation to adjust for signal changes in volumes during spatial normalization, and (iii) spatial smoothing of the gray matter partitions using a Gaussian kernel (6 mm full-width at half maximum). After the preprocessing, rGVMs were extracted by averaging the values of the 116 brain regions according to the AAL atlas (Desikan et al., 2006). To minimize the effects of potential nuisance covariates, we regressed out the linear effects of age, sex, total intracranial volume, and years of education from each of the rGMV values.

### Extraction of Activity Characteristics

**FIGURE 2:**
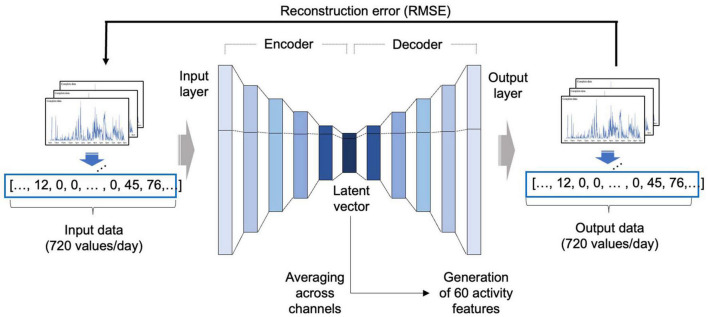
Development of the autoencoder model. An autoencoder consists of an encoder that receives input data and compresses the information into a latent feature, and a decoder that receives the compressed latent feature and restores the original input in the direction that minimizes the RMSE. In this study, after autoencoder training using the NHANES data, a latent vector was obtained from the activity dataset of the BICWALZS by applying the encoder portion of the autoencoder and averaging across channels to obtain 60 activity features. RMSE, root mean squared error; NHANES, National Health and Nutrition Examination Survey; BICWALZS, Biobank Innovations for chronic Cerebrovascular disease With ALZheimer’s disease Study.

Since the activity data were 1-dimensional data measured continuously over time, a 1-dimensional convolutional autoencoder was adopted to reflect the temporal characteristics of the activity data (Hinton and Salakhutdinov, 2006; Figure 2).

The basic structure and initial hyperparameter values were based on our previous model (Jang et al., 2020). The model development was conducted based on the NHANES activity dataset, which was randomly divided into portions at ratios of 8:1:1 for use as a training set, a validation set, and a test set, respectively. The autoencoder was trained using the training set, and the hyperparameters were tuned using the validation set. Root-mean-square error (RMSE) was adopted as the cost function. The Adam optimization algorithm was used as the optimizer, along with the ReLU activation function.

After model development, we extracted the latent vector from the BICWALZS activity dataset using the encoder portion of the autoencoder. All channels in the extracted latent vector were averaged to create 60 activity features. When one patient had recorded data over several days, the overall average of the extracted activity features from each day was employed as the representative value of the activity features.

The overview of the autoencoder-based feature extraction is described in Figure 2. Detailed information on the selected autoencoder model is described in Supplementary Table 2, and the detailed results of the visualization are described in Supplementary Figure 1.

To extract time-frequency features, this study additionally applied the short-time Fourier transform (STFT) which was performed by convolving a short-time squared window function with non-stationary assumed activity time series. Each STFT window was Fourier-transformed with the following parameters: total time points = 720 (inter-sample interval = 1 min); sampling frequency = 0.0167 (1 sample per 1 min); and window size = 60 min. For this, we used a MATLAB function, “spectrogram.m,” with default options and estimated magnitude for each frequency and averaged them over all time points. To avoid redundant information over all frequencies, we grouped all frequencies’ indices (= 128) to 64 with the same bins and again averaged the averaged magnitudes within each.

### Association Analysis Between Activity Features and Brain Volume

#### Brain Region Level Analysis

To estimate the association between activity features and regional brain volume, cross-sectional values for the 116 regional brain volumes (dependent variables) were linearly projected onto all activity features (independent variables). Backward elimination procedures were used to select for the meaningful features in each regression model separately (Gheyas and Smith, 2010). Subsequently, we extracted each regression model’s r-squared values and calculated the Pearson correlation coefficient between the estimated brain volume from the regression model and the actual brain volume.

Association estimates (i.e., regression coefficients) from regression models were considered significant at a *P* value < 0.05. To address multiple comparison issues when assessing the significance of 116 regression models, the Benjamini-Hochberg Procedure was employed to control for the false discovery rate (FDR), and only models with an FDR of < 0.05 were considered statistically significant. Regression models with an FDR value > 0.05 were considered statistically insignificant and were excluded from the analysis.

To compare our deep learning-based activity features characteristics with those of features derived by traditional frequency domain feature extraction method, we fitted and compared two regression models: (1) one employing both deep learning-based features and frequency domain features; (2) one based only on the frequency domain features.

#### Lobe Level Analysis

We also grouped 116 individual regions based on brain lobes according to the AAL atlas (Desikan et al., 2006). As a result, each region was classified as belonging to the limbic, temporal, parietal, occipital, frontal, subcortical, central, or cerebellar lobe. The list of regions grouped by each lobe is summarized in Multimedia Supplementary Table 3.

The distributions of the regional correlations within each lobe were illustrated using violin plots, and we tabulated the average regional correlation coefficients and the average r-squared values within each lobe.

### Software Tools

In our study, the scripts used for the preprocessing of the accelerometer data were written in Python version 3.7 using the scikit-learn and statsmodel Python packages. We used the PyTorch framework to construct a deep learning model. For visualization, the Matplotlib and Seaborn Python packages were used. All statistical analyses relating to the brain MRI data were performed using MATLAB (MathWorks, Sherborn, MA, United States) based custom software.

## Results

After preprocessing, the NHANES dataset comprised 25,858 day-long sets of physical activity data from 9,236 individuals, whereas the BICWALZS dataset comprised 528 day-long sets of data from 132 individuals.

Because the NHANES dataset was based on data collected from the general population, in contrast with the BICWALZS dataset, which was generated from patients with dementia-related symptoms, the demographic characteristics were different. Individuals in the BICWALZS dataset were older, with a lower body mass index and had a dementia-related diagnosis. Detailed baseline characteristics for each dataset are summarized in Table 1.

**TABLE 1:** Baseline characteristics of the two datasets.

Characteristics		NHANES dataset (*n* = 14,482)	BICWALZS dataset (*n* = 177)	*P* values
Age (years), mean (SD)		39.04 (22.27)	74.07 (7.05)	<0.001
**Sex**				
Male, n (%)		7,055 (48.71)	56 (31.6)	
Female, n (%)		7,427 (51.28)	121 (68.3)	
Weight (kg), mean (SD)		75.26 (21.73)	59.03 (10.04)	<0.001
Height (cm), mean (SD)		166.01 (11.72)	156.96 (8.33)	<0.001
BMI (kg/m^2^), mean (SD)		27.03 (6.56)	22.66 (7.19)	<0.001
Device		Actigraph AM-7164 (uniaxial)	FitNLife Fitmeter (triaxial)	
Education (years), mean (SD)		–	9.14 (4.56)	–
**Main diagnosis at time of visit**				
	Subjective memory loss, n (%)	–	18 (10.2)	–
	Mild cognitive impairment, n (%)	–	89 (50.3)	–
	Alzheimer’s disease, n (%)	–	47 (26.6)	–
	Subcortical vascular dementia, n (%)	–	14 (8.9)	–
	Frontotemporal dementia, n (%)	–	4 (2.2)	–
	Other, n (%)	–	4 (2.2)	
MMSE score, mean (SD)		–	23.17 (6.33)	–
CDR score, mean (SD)		–	0.76 (0.48)	–
GDS score, mean (SD)			5.53 (4.45)	

*NHANES, National Health and Nutrition Examination Survey dataset; BICWALZS, Biobank Innovations for chronic Cerebrovascular disease With ALZheimer’s disease Study; BMI, body mass index; SD, standard deviation; MMSE, Mini-Mental State Examination, CDR, Clinical Dementia Rating, GDS, Global Deterioration Scale.*

### Consistency Between the Estimated and Actual Brain Volumes

**FIGURE 3:**
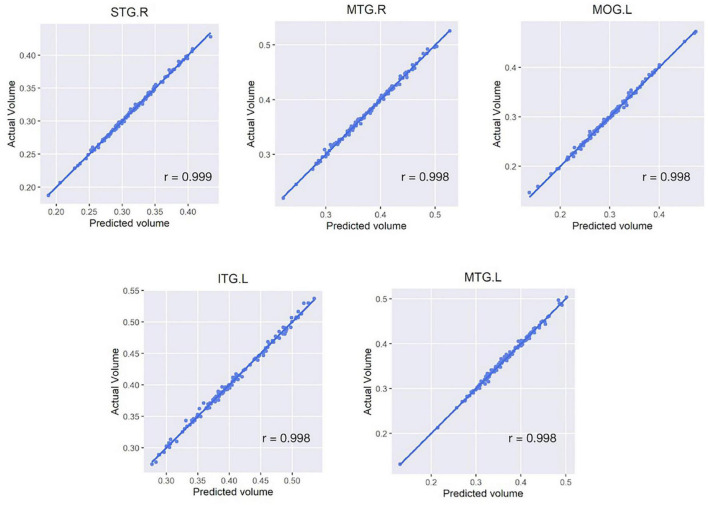
Scatter plots of five regions with the highest correlation between the actual and estimated volumes. Five regions include the right superior/middle temporal gyrus, left inferior/middle temporal gyrus, and left middle occipital gyrus. For all subfigures, the y-axis represents the actual brain volume and the x-axis represents the estimated brain volume based on the linear regression model generated from the features extracted from the activity data. STG, superior temporal gyrus; MTG, middle temporal gyrus; MOG, middle occipital gyrus; ITG, inferior temporal gyrus; L/R, left/right hemisphere.

In each regional analysis, mean Pearson correlation coefficient in all areas was 0.990 (mean r-squared value 0.979), with all values > 0.964. Four of five regions showing the highest correlations were located in the temporal lobe: the right superior/middle temporal gyrus and left inferior/middle temporal gyrus (Figure 3). The mean correlation coefficient for those five regions was 0.998, and the mean r-squared value for the five estimation models was 0.996. When both deep learning-based features and time-frequency domain features were used, the mean Pearson correlation coefficient was significantly greater than that when only time-frequency domain features were used (0.990 vs. 0.860).

To address the multiple comparison problem, the Benjamini-Hochberg Procedure was employed to control for the FDR. The FDR values were calculated for each of the 116 correlations. Consequently, it was observed that the FDR was < 0.05 in all the regression models. The correlation coefficients values for each region are detailed in Supplementary Table 4.

**FIGURE 4:**
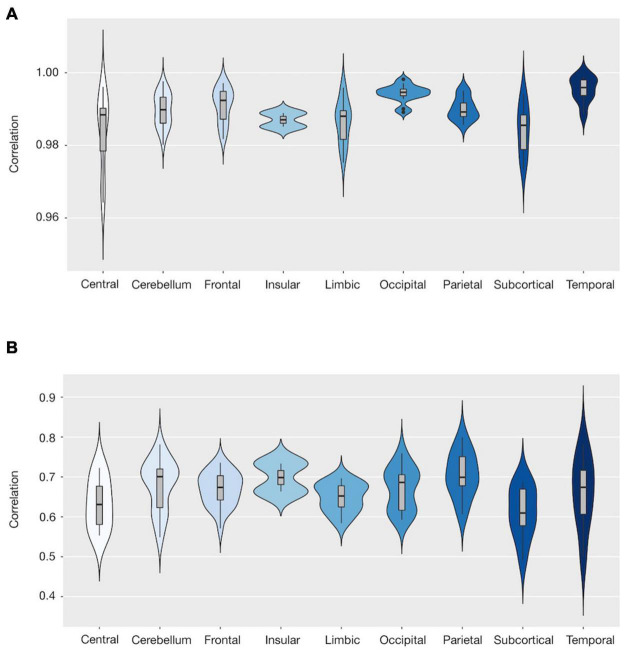
Violin plot showing the distribution of the correlations between the actual and estimated volumes by region within each lobe. We present two results: from combining deep learning and frequency features **(A)**, and from frequency features solely **(B)**. The X-axis shows the lobe defined according to the automated anatomical labeling (AAL) atlas, whereas the Y-axis shows the correlation coefficients for each lobe. The broader sections of the violin plot represent a higher probability, whereas the thinner sections represent a lower probability. The thick black bar in the center represents the interquartile range, whereas the thin black line represents 1.5 times the interquartile range.

We also examined the correlations between the actual and expected volumes at a larger spatial scale at the lobe level. Each lobe showed a consistent level of correlation. The highest correlation was observed in the temporal lobe (*r* = 0.995), followed by the occipital (*r* = 0.994), frontal (*r* = 0.991), and parietal and cerebellar (*r* = 0.99) lobes (Table 2 and Figure 4).

**TABLE 2:** Correlation between actual and expected volumes for each lobe.

	Deep learning + Time-frequency domain features			Time-frequency domain features only			*P* value^†^	*P* value^‡^
Lobe	Number of regions (excluded regions)	Mean correlation (SD)	Mean r^2^ value (SD)	Number of regions (excluded regions)	Mean correlation (SD)	Mean r^2^ value (SD)		
Central	8 (2)	0.989 (0.01)	0.979 (0.01)	8 (0)	0.804 (0.09)	0.654 (0.13)	0.006	0.002
Cerebellum	18 (0)	0.99 (0)	0.979 (0.01)	18 (0)	0.799 (0.04)	0.64 (0.07)	<0.001	<0.001
Frontal	24 (0)	0.991 (0)	0.982 (0.01)	24 (0)	0.896 (0.02)	0.803 (0.04)	<0.001	<0.001
Insula	2 (0)	0.987 (0)	0.974 (0.01)	2 (0)	0.914 (0)	0.835 (0)	0.034	0.043
Limbic	14 (0)	0.986 (0.01)	0.973 (0.01)	14 (0)	0.867 (0.03)	0.753 (0.05)	<0.001	<0.001
Occipital	14 (0)	0.994 (0)	0.989 (0)	14 (0)	0.899 (0.03)	0.81 (0.06)	<0.001	<0.001
Parietal	10 (0)	0.99 (0)	0.98 (0.01)	10 (0)	0.883 (0.02)	0.779 (0.03)	<0.001	<0.001
Subcortical	10 (0)	0.984 (0.01)	0.969 (0.01)	10 (0)	0.809 (0.05)	0.657 (0.08)	<0.001	<0.001
Temporal	8 (0)	0.995 (0)	0.991 (0.01)	8 (0)	0.918 (0.02)	0.843 (0.03)	<0.001	<0.001

*^†^Difference in mean correlation (t-test). ^‡^Difference in mean r^2^ (t-test). ^†,‡^Adjusted by Bonferroni correction.*

## Discussion

In this study, we attempted to extract meaningful features from patients’ physical activity data using a deep learning model, and we showed that these data could be used for predicting brain volume. Consequently, we generated a statistically significant brain volume estimation model for 116 regions, which were anatomically subdivided according to the AAL atlas. The mean correlation between the actual and estimated volumes of the regression models for these 116 regions was 0.990.

In the lobe-scale analysis performed combining deep learning and frequency features, the temporal lobe demonstrated the highest mean correlation (0.995), followed by the occipital (0.994), and the frontal, parietal, and cerebellar lobes. Our approach showed an excellent prediction performance for all brain lobes (mean correlation ≥ 0.984 at least).

While the numbers of patients with dementia and MCI are rapidly increasing due to recent changes in the proportions of aging populations, only a fraction of patients with MCI progress to clinical AD (Visser et al., 2006; Mitchell, 2009). According to two community-based studies, over a third of MCI patients recovered normal cognition (Larrieu et al., 2002; Ganguli et al., 2004), although examining the risk of dementia progression in the early stage of cognitive dysfunction can still lead to appropriate intervention. Brain atrophy in limbic regions, such as the hippocampus, parahippocampal gyrus, and amygdala, is known to be a significant predictor of progression to AD (Korf et al., 2004; DeCarli et al., 2007), and brain MRI is the current gold standard for examining brain atrophy; however, due to the expense and medical device requirements, continuous MRI monitoring is difficult, as is early detection because brain MRI does not typically occur before signs of the disease begin manifesting. Therefore, a cost-effective, continuous, and easily accessible alternative screening tool is required.

The correlation between brain volume atrophy and the amount of brain activity has already been studied (Colcombe et al., 2006; Ploughman, 2008), and the increasingly widespread use of wearable devices has made it easier to measure such activity accurately. Most commercial smartwatches can now be purchased for less than 500 USD, which is cheaper than the cost of a single MRI examination, and there is a huge demand for the purchase of such devices, even without the need for medical care. In addition, because mobile devices can monitor the subjects continuously in real-time, they have some inherent and unique advantages compared with conventional screening tools (e.g., MRI or survey) for detecting dementia.

Our findings suggest that deep learning-based and time-frequency domain features independently provide meaningful information on brain volume. We observed performance improvement for brain volume prediction when using both deep learning-based features and time-frequency domain features. In addition, using an external dataset to train the deep learning-based features increased the generalizability power of the model. As both autoencoder model structure (Supplementary Table 2) and data are open access, any researcher will be able to extract the same activity features used in this study and repurpose them for their own research.

Several brain volume estimation studies have been conducted using an accelerometer. For example, Arnardottir et al. studied the association between changes in brain structure and accelerometer-measured physical activity (Arnardottir et al., 2016), and Spartano et al. (2019) investigated the association between accelerometer-measured low-intensity physical activity and brain volume. Tan et al. (2017) also studied the relationship between physical activity and brain volume, particularly in a region specific to dementia, the hippocampus. However, these previous studies used simple statistics like total daily activity as the representative measure of the activity features, making it difficult to utilize the temporal, non-linear pattern of the activity data. Also, the brain volume estimations have been limited to estimating the total brain volume or the volumes of only a few regions. Crucially, previous models have had to use dozens of clinical variables to estimate brain volume, making these methods ill-suited for out-of-hospital mobile environments where it is difficult to obtain clinical information.

Unlike previous studies, we solely employed activity features that were extracted by an unsupervised deep learning model and a time-frequency domain feature extraction model to estimate brain volumes. Although we did not include clinical information in our model, it demonstrated good performance in estimating brain volumes; we thus believe that our feature extraction method captures information intrinsic to activity data that contributed to this performance. Recently, several studies have been successful in estimating clinical information that was not explicitly provided, such as in the prediction of valve disease or hyperkalemia using deep learning in electrocardiogram studies (Galloway et al., 2019; Kwon et al., 2020). This supports the hypothesis that hidden but clinically meaningful information may exist in bio-signals, and these patterns could be analyzed with deep learning methods (Yoon et al., 2020).

We were the first to develop statistically significant estimation models for the volume of 116 brain regions. At the lobe level, the estimation power for temporal lobe atrophy, which is associated with memory and executive functions, as well as with MCI and AD (Guillozet et al., 2003; Nho et al., 2012), was the highest among all the lobes. To develop a reliable screening test, further detailed studies of brain sub-regions are needed.

One of the strengths of this study was that it was based on multi-centered, multinational datasets. The United States NHANES dataset, based on data collected from the general population, was used for training, and the Korean tertiary center dataset, based on patients with cognitive dysfunction, was used for validating the model externally. This strategy showed that the conclusion based on the extracted activity features in our model could be generalized across data from different nations and populations.

Assessments of cognitive function in the BICWALZS confirmed that the model developed in the study was able to detect the reduced volume of brain regions using physical activity patterns, even in those in the early stage of dementia. In this population, 60.5% of subjects had complained only of subjective memory loss or MCI. The average Mini-Mental State Examination (MMSE) score in these subjects was 23.17, which was close to the 24-point cut-off used as the general boundary for dementia screening (Kang et al., 1997). In patients with dementia, the Clinical Dementia Rating (CDR) score was 0.76 on average, which is considered to be indicative of mild dementia (Morris, 1991).

There were some limitations to this study that should be addressed. Firstly, although we adopted a deep learning method for generating the feature extraction model, a classical linear regression model was employed in the estimation process using activity features because we could not secure a large enough sample size to allow us to apply the deep learning approach. Secondly, the low number of subjects hindered our ability to test the brain volume estimation model using an independent dataset. Therefore, this study focused on evaluating the usefulness of assessing those features by estimating the correlations between the expected and actual brain volumes. To improve the performance of the brain volume estimation method and to ensure its generalizability, we could apply the latest machine learning developments, such as the use of a deep neural network and conduct a test with an external dataset in a future study with a larger patient sample size. Third, we did not collect MRI images and activity data serially for a long period. Therefore, further study with time-series data is required to discover whether our approach could be effective in detecting and monitoring changes of brain volume.

In conclusion, the results of this study suggest that activity features from activity data can be successfully used to estimate brain volume changes, even in patients with mild neurodegenerative symptoms. Because our model relied solely on the activity features extracted from accelerometer data, we could minimize the requirement for clinical information. We expect that our model will contribute to the detection of dementia or other brain volume-related diseases, especially in a mobile environment.

## Data Availability Statement

The datasets presented in this article are not readily available because requests to access them must first be approved by the Ajou University Hospital Institutional Review Board. Requests to access the datasets should be directed to SS, sjsonpsy@ajou.ac.kr.

## Ethics Statement

Written informed consent was obtained from the individual(s) for the publication of any potentially identifiable images or data included in this article.

## Author Contributions

BP, BC, EK, and DY contributed to conceptualization of the research. HL, J-HJ, and HR contributed to statistical analysis and programming. CH and SS acquired funding. BC prepared original draft. BP and DY made critical revisions to the manuscript. All authors revised the manuscript and approved the final version of the manuscript.

## Conflict of Interest

DY is the founder and employee of BUD.on Inc. The remaining authors declare that the research was conducted in the absence of any commercial or financial relationships that could be construed as a potential conflict of interest.

## Publisher’s Note

All claims expressed in this article are solely those of the authors and do not necessarily represent those of their affiliated organizations, or those of the publisher, the editors and the reviewers. Any product that may be evaluated in this article, or claim that may be made by its manufacturer, is not guaranteed or endorsed by the publisher.

## Footnotes

1
http://www.fil.ion.ucl.ac.uk/spm/
