# Supplementary material for: Modeling Brain Volume Using Deep Learning-Based Physical Activity Features in Patients With Dementia
Source: Front Neuroinform. 2022 Mar 9;16:795171. doi: 10.3389/fninf.2022.795171 (PMC8959707; doi:10.3389/fninf.2022.795171)
Supplement: Supplementary file 1 [file Table_1.DOCX]

# Supplementary Table 1. Detailed information about the accelerometer devices

| Characteristic | NHANES dataset | BICWALZS dataset |
| --- | --- | --- |
| Device | Actigraph AM-7164 | Fit.Life Fitmeter |
| Manufacturer | Actigraph | FitNLife |
| Sampling Rate | 1 minute | 10 seconds |
| Accelerometer value (cpm), mean (SD) | 344 (694.23) | 637 (1,121.27) |
| Wearable location | Wrist | Right hip |
| Number of axes | Uniaxial (Z-axis) | Triaxial (X-, Y-, and Z-axes) |

NHANES, National Health and Nutrition Examination Survey;

BICWALZS, Biobank Innovations for Chronic Cerebrovascular Disease with ALZheimer's Disease Study; cpm, counts per minute; SD, standard deviation.
