# Supplementary material for: Modeling Brain Volume Using Deep Learning-Based Physical Activity Features in Patients With Dementia
Source: Front Neuroinform. 2022 Mar 9;16:795171. doi: 10.3389/fninf.2022.795171 (PMC8959707; doi:10.3389/fninf.2022.795171)
Supplement: Supplementary file 2 [file Table_2.DOCX]

# Supplementary Table 2. Structure of the convolutional autoencoder used in the modeling

| Component | Layer name  (order of layer) | Filter  n x size (stride) | Output size  Feature map (n x size) |
| --- | --- | --- | --- |
|  |  |  |  |
| **Input** |  |  |  |
|  |  |  | 1 × 720 |
| **Encoder** |  |  |  |
|  | Convolution (1) | 8×30 (2) | 8 × 346 |
|  | Convolution (2) | 16 × 20 (2) | 16 × 164 |
|  | Convolution (3) | 32 × 10 (2) | 32 × 78 |
|  | Convolution (4) | 64 × 10 (1) | 64 × 69 |
|  | Convolution (5) | 128 × 10 (1) | 128 × 60^a^ |
|  | Convolution (6) | 64 × 1 (1) | 64 × 60^a^ |
| **Decoder** |  |  |  |
|  | Transconvolution (6) | 64 × 10 (1) | 64 × 69 |
|  | Transconvolution (7) | 32 × 10 (1) | 32 × 78 |
|  | Transconvolution (8) | 16 × 10 (2) | 16 × 164 |
|  | Transconvolution (9) | 8 × 20 (2) | 8 × 346 |
|  | Transconvolution(10) | 1 × 30 (2) | 1 × 720 |

In this study, we adopted a convolutional autoencoder. The model structure of the autoencoder is described above. ^a^Latent vector size extracted by the encoder; n, the number of filters.
