# Supplementary material for: Modeling Brain Volume Using Deep Learning-Based Physical Activity Features in Patients With Dementia
Source: Front Neuroinform. 2022 Mar 9;16:795171. doi: 10.3389/fninf.2022.795171 (PMC8959707; doi:10.3389/fninf.2022.795171)
Supplement: Supplementary file 3 [file Table_3.DOCX]

# Supplementary Table 3. The list of brain regions grouped by each lobe

| Region | Lobe |
| --- | --- |
| Paracentral_Lobule_L | central |
| Paracentral_Lobule_R | central |
| Postcentral_L | central |
| Postcentral_R | central |
| Precentral_L | central |
| Precentral_R | central |
| Rolandic_Oper_L | central |
| Rolandic_Oper_R | central |
| Cerebelum_10_L | cerebellum |
| Cerebelum_10_R | cerebellum |
| Cerebelum_3_L | cerebellum |
| Cerebelum_3_R | cerebellum |
| Cerebelum_4_5_L | cerebellum |
| Cerebelum_4_5_R | cerebellum |
| Cerebelum_6_L | cerebellum |
| Cerebelum_6_R | cerebellum |
| Cerebelum_7b_L | cerebellum |
| Cerebelum_7b_R | cerebellum |
| Cerebelum_8_L | cerebellum |
| Cerebelum_8_R | cerebellum |
| Cerebelum_9_L | cerebellum |
| Cerebelum_9_R | cerebellum |
| Cerebelum_Crus1_L | cerebellum |
| Cerebelum_Crus1_R | cerebellum |
| Cerebelum_Crus2_L | cerebellum |
| Cerebelum_Crus2_R | cerebellum |
| Vermis_1_2 | cerebellum |
| Vermis_10 | cerebellum |
| Vermis_3 | cerebellum |
| Vermis_4_5 | cerebellum |
| Vermis_6 | cerebellum |
| Vermis_7 | cerebellum |
| Vermis_8 | cerebellum |
| Vermis_9 | cerebellum |
| Frontal_Inf_Oper_L | frontal |
| Frontal_Inf_Oper_R | frontal |
| Frontal_Inf_Orb_L | frontal |
| Frontal_Inf_Orb_R | frontal |
| Frontal_Inf_Tri_L | frontal |
| Frontal_Inf_Tri_R | frontal |
| Frontal_Med_Orb_L | frontal |
| Frontal_Med_Orb_R | frontal |
| Frontal_Mid_L | frontal |
| Frontal_Mid_Orb_L | frontal |
| Frontal_Mid_Orb_R | frontal |
| Frontal_Mid_R | frontal |
| Frontal_Sup_L | frontal |
| Frontal_Sup_Medial_L | frontal |
| Frontal_Sup_Medial_R | frontal |
| Frontal_Sup_Orb_L | frontal |
| Frontal_Sup_Orb_R | frontal |
| Frontal_Sup_R | frontal |
| Olfactory_L | frontal |
| Olfactory_R | frontal |
| Rectus_L | frontal |
| Rectus_R | frontal |
| Supp_Motor_Area_L | frontal |
| Supp_Motor_Area_R | frontal |
| Insula_L | Insula |
| Insula_R | Insula |
| Cingulum_Ant_L | limbic |
| Cingulum_Ant_R | limbic |
| Cingulum_Mid_L | limbic |
| Cingulum_Mid_R | limbic |
| Cingulum_Post_L | limbic |
| Cingulum_Post_R | limbic |
| Hippocampus_L | limbic |
| Hippocampus_R | limbic |
| ParaHippocampal_L | limbic |
| ParaHippocampal_R | limbic |
| Temporal_Pole_Mid_L | limbic |
| Temporal_Pole_Mid_R | limbic |
| Temporal_Pole_Sup_L | limbic |
| Temporal_Pole_Sup_R | limbic |
| Calcarine_L | occipital |
| Calcarine_R | occipital |
| Cuneus_L | occipital |
| Cuneus_R | occipital |
| Fusiform_L | occipital |
| Fusiform_R | occipital |
| Lingual_L | occipital |
| Lingual_R | occipital |
| Occipital_Inf_L | occipital |
| Occipital_Inf_R | occipital |
| Occipital_Mid_L | occipital |
| Occipital_Mid_R | occipital |
| Occipital_Sup_L | occipital |
| Occipital_Sup_R | occipital |
| Angular_L | parietal |
| Angular_R | parietal |
| Parietal_Inf_L | parietal |
| Parietal_Inf_R | parietal |
| Parietal_Sup_L | parietal |
| Parietal_Sup_R | parietal |
| Precuneus_L | parietal |
| Precuneus_R | parietal |
| SupraMarginal_L | parietal |
| SupraMarginal_R | parietal |
| Amygdala_L | subcortical |
| Amygdala_R | subcortical |
| Caudate_L | subcortical |
| Caudate_R | subcortical |
| Pallidum_L | subcortical |
| Pallidum_R | subcortical |
| Putamen_L | subcortical |
| Putamen_R | subcortical |
| Thalamus_L | subcortical |
| Thalamus_R | subcortical |
| Heschl_L | temporal |
| Heschl_R | temporal |
| Temporal_Inf_L | temporal |
| Temporal_Inf_R | temporal |
| Temporal_Mid_L | temporal |
| Temporal_Mid_R | temporal |
| Temporal_Sup_L | temporal |
| Temporal_Sup_R | temporal |
