# Supplementary material for: Modeling Brain Volume Using Deep Learning-Based Physical Activity Features in Patients With Dementia
Source: Front Neuroinform. 2022 Mar 9;16:795171. doi: 10.3389/fninf.2022.795171 (PMC8959707; doi:10.3389/fninf.2022.795171)
Supplement: Supplementary file 4 [file Table_4.DOCX]

# Supplementary Table 4. Correlation analysis results of all brain regions

| Region | Deep learning + Time-frequency domain features | | Time-frequency domain features only | |
| --- | --- | --- | --- | --- |
|  | Correlation between actual volume and estimated volume | R-squared value of estimated regression model | Correlation between actual volume and estimated volume | R-squared value of estimated regression model |
| Precentral_L | 0.987992752 | 0.976129678 | 0.791246191 | 0.626070536 |
| Precentral_R | 0.992831447 | 0.985714282 | 0.827835924 | 0.685312317 |
| Frontal_Sup_L | 0.991734535 | 0.983537388 | 0.895233868 | 0.801443678 |
| Frontal_Sup_R | 0.997078775 | 0.994166083 | 0.909534736 | 0.827253436 |
| Frontal_Sup_Orb_L | 0.993337852 | 0.986720089 | 0.932718664 | 0.869964106 |
| Frontal_Sup_Orb_R | 0.997147497 | 0.994303132 | 0.904309248 | 0.817775215 |
| Frontal_Mid_L | 0.993339928 | 0.986724212 | 0.881389036 | 0.776846633 |
| Frontal_Mid_R | 0.99262497 | 0.98530433 | 0.882246351 | 0.778358625 |
| Frontal_Mid_Orb_L | 0.987340486 | 0.974841236 | 0.912608179 | 0.832853688 |
| Frontal_Mid_Orb_R | 0.996357122 | 0.992727514 | 0.898611584 | 0.807502778 |
| Frontal_Inf_Oper_L | 0.992971263 | 0.98599193 | 0.868382188 | 0.754087624 |
| Frontal_Inf_Oper_R | 0.997034756 | 0.994078306 | 0.862729304 | 0.744301852 |
| Frontal_Inf_Tri_L | 0.991035257 | 0.982150881 | 0.890535557 | 0.793053579 |
| Frontal_Inf_Tri_R | 0.995063723 | 0.990151813 | 0.903074325 | 0.815543237 |
| Frontal_Inf_Orb_L | 0.981737757 | 0.963809024 | 0.880043593 | 0.774476726 |
| Frontal_Inf_Orb_R | 0.993515733 | 0.987073511 | 0.868284974 | 0.753918797 |
| Rolandic_Oper_L | 0.989358306 | 0.978829857 | 0.864241476 | 0.746913329 |
| Rolandic_Oper_R | 0.996111752 | 0.992238623 | 0.898530458 | 0.807356985 |
| Supp_Motor_Area_L | 0.995620172 | 0.991259527 | 0.880232371 | 0.774809026 |
| Supp_Motor_Area_R | 0.985379463 | 0.970972686 | 0.880030889 | 0.774454365 |
| Olfactory_L | 0.992126504 | 0.984314999 | 0.925252257 | 0.856091739 |
| Olfactory_R | 0.982922169 | 0.966135991 | 0.876971927 | 0.769079761 |
| Frontal_Sup_Medial_L | 0.987001755 | 0.974172464 | 0.925788042 | 0.857083499 |
| Frontal_Sup_Medial_R | 0.987266227 | 0.974694603 | 0.882509813 | 0.778823569 |
| Frontal_Med_Orb_L | 0.982779066 | 0.965854693 | 0.915295891 | 0.837766568 |
| Frontal_Med_Orb_R | 0.985904912 | 0.972008495 | 0.882275737 | 0.778410477 |
| Rectus_L | 0.990360117 | 0.98081316 | 0.92346272 | 0.852783395 |
| Rectus_R | 0.994793646 | 0.989614398 | 0.915935602 | 0.838938026 |
| Insula_L | 0.985153848 | 0.970528104 | 0.912557948 | 0.832762008 |
| Insula_R | 0.988895637 | 0.977914582 | 0.914464485 | 0.836245294 |
| Cingulum_Ant_L | 0.989244644 | 0.978604966 | 0.87915555 | 0.772914481 |
| Cingulum_Ant_R | 0.975210265 | 0.951035061 | 0.832178643 | 0.692521294 |
| Cingulum_Mid_L | 0.995875844 | 0.991768697 | 0.898858376 | 0.80794638 |
| Cingulum_Mid_R | 0.995365679 | 0.990752834 | 0.920449264 | 0.847226847 |
| Cingulum_Post_L | 0.988073982 | 0.976290194 | 0.874911083 | 0.765469403 |
| Cingulum_Post_R | 0.985721158 | 0.971646202 | 0.896932601 | 0.804488091 |
| Hippocampus_L | 0.991136075 | 0.982350718 | 0.888846405 | 0.790047931 |
| Hippocampus_R | 0.98453959 | 0.969318204 | 0.863373948 | 0.745414574 |
| ParaHippocampal_L | 0.989640181 | 0.979387688 | 0.883912141 | 0.781300673 |
| ParaHippocampal_R | 0.987968137 | 0.976081041 | 0.851611079 | 0.725241429 |
| Amygdala_L | 0.993098936 | 0.986245497 | 0.860088412 | 0.739752076 |
| Amygdala_R | 0.98685258 | 0.973878016 | 0.838578554 | 0.703213991 |
| Calcarine_L | 0.995124787 | 0.990273341 | 0.887784838 | 0.788161919 |
| Calcarine_R | 0.99409476 | 0.988224392 | 0.88287188 | 0.779462756 |
| Cuneus_L | 0.993964263 | 0.987964956 | 0.925688002 | 0.856898276 |
| Cuneus_R | 0.995148426 | 0.990320389 | 0.916646289 | 0.840240418 |
| Lingual_L | 0.990054791 | 0.980208489 | 0.93206937 | 0.868753311 |
| Lingual_R | 0.995106332 | 0.990236611 | 0.920670915 | 0.847634934 |
| Occipital_Sup_L | 0.993788637 | 0.987615855 | 0.902187094 | 0.813941553 |
| Occipital_Sup_R | 0.992792202 | 0.985636356 | 0.873819245 | 0.763560073 |
| Occipital_Mid_L | 0.998185654 | 0.996374601 | 0.8916414 | 0.795024386 |
| Occipital_Mid_R | 0.995574242 | 0.991168072 | 0.87116567 | 0.758929624 |
| Occipital_Inf_L | 0.99604716 | 0.992109945 | 0.873432118 | 0.762883664 |
| Occipital_Inf_R | 0.989128229 | 0.978374653 | 0.835420118 | 0.697926774 |
| Fusiform_L | 0.997071899 | 0.994152371 | 0.92759631 | 0.860434915 |
| Fusiform_R | 0.99362496 | 0.987290562 | 0.948813911 | 0.900247838 |
| Postcentral_L | 0.981067361 | 0.962493167 | 0.854968075 | 0.73097041 |
| Postcentral_R | 0.988820894 | 0.977766759 | 0.844906257 | 0.713866584 |
| Parietal_Sup_L | 0.98788403 | 0.975914857 | 0.86818884 | 0.753751862 |
| Parietal_Sup_R | 0.988146002 | 0.976432522 | 0.864437261 | 0.747251779 |
| Parietal_Inf_L | 0.990221314 | 0.98053825 | 0.889960975 | 0.792030537 |
| Parietal_Inf_R | 0.988125165 | 0.976391342 | 0.872706127 | 0.761615983 |
| SupraMarginal_L | 0.991730786 | 0.983529952 | 0.883909747 | 0.781296441 |
| SupraMarginal_R | 0.991420615 | 0.982914836 | 0.917969165 | 0.842667388 |
| Angular_L | 0.985630684 | 0.971467844 | 0.872254683 | 0.760828233 |
| Angular_R | 0.99493154 | 0.989888769 | 0.882038978 | 0.777992758 |
| Precuneus_L | 0.995042633 | 0.990109842 | 0.896919238 | 0.80446412 |
| Precuneus_R | 0.987628544 | 0.97541014 | 0.876731547 | 0.768658206 |
| Paracentral_Lobule_L | 0.964228301 | 0.929736216 | 0.632258241 | 0.399750483 |
| Paracentral_Lobule_R | 0.970561487 | 0.941989599 | 0.721724111 | 0.520885693 |
| Caudate_L | 0.981405511 | 0.963156776 | 0.871961955 | 0.760317651 |
| Caudate_R | 0.973303534 | 0.94731977 | 0.770982658 | 0.594414259 |
| Putamen_L | 0.994091978 | 0.988218861 | 0.811284064 | 0.658181832 |
| Putamen_R | 0.984200564 | 0.968650751 | 0.783235865 | 0.61345842 |
| Pallidum_L | 0.977999924 | 0.95648385 | 0.756886854 | 0.57287771 |
| Pallidum_R | 0.976420215 | 0.953396436 | 0.705004247 | 0.497030988 |
| Thalamus_L | 0.988808673 | 0.977742593 | 0.848148951 | 0.719356643 |
| Thalamus_R | 0.98703485 | 0.974237796 | 0.841810583 | 0.708645058 |
| Heschl_L | 0.988516278 | 0.977164432 | 0.898980086 | 0.808165195 |
| Heschl_R | 0.993926006 | 0.987888905 | 0.904152364 | 0.817491497 |
| Temporal_Sup_L | 0.994159788 | 0.988353683 | 0.921389011 | 0.84895771 |
| Temporal_Sup_R | 0.998983361 | 0.997967755 | 0.927240528 | 0.859774998 |
| Temporal_Pole_Sup_L | 0.988245518 | 0.976629204 | 0.8310149 | 0.690585764 |
| Temporal_Pole_Sup_R | 0.979865944 | 0.960137269 | 0.848836863 | 0.720524021 |
| Temporal_Mid_L | 0.997652867 | 0.995311242 | 0.923170589 | 0.852243936 |
| Temporal_Mid_R | 0.998411033 | 0.996824591 | 0.948958438 | 0.900522117 |
| Temporal_Pole_Mid_L | 0.980685924 | 0.961744881 | 0.82582898 | 0.681993504 |
| Temporal_Pole_Mid_R | 0.979356429 | 0.959139015 | 0.843009465 | 0.710664958 |
| Temporal_Inf_L | 0.997859576 | 0.995723734 | 0.896845914 | 0.804332593 |
| Temporal_Inf_R | 0.993158604 | 0.986364013 | 0.92159596 | 0.849339113 |
| Cerebelum_Crus1_L | 0.988253759 | 0.976645491 | 0.773036721 | 0.597585772 |
| Cerebelum_Crus1_R | 0.985111856 | 0.970445368 | 0.782980734 | 0.61305883 |
| Cerebelum_Crus2_L | 0.993378681 | 0.986801204 | 0.785538097 | 0.617070102 |
| Cerebelum_Crus2_R | 0.99201709 | 0.984097906 | 0.761948552 | 0.580565596 |
| Cerebelum_3_L | 0.991607018 | 0.983284478 | 0.813527804 | 0.661827488 |
| Cerebelum_3_R | 0.99240691 | 0.984871475 | 0.77082221 | 0.59416688 |
| Cerebelum_4_5_L | 0.985904986 | 0.972008642 | 0.798866438 | 0.638187586 |
| Cerebelum_4_5_R | 0.990234076 | 0.980563526 | 0.814701204 | 0.663738051 |
| Cerebelum_6_L | 0.984927262 | 0.970081711 | 0.831777749 | 0.691854224 |
| Cerebelum_6_R | 0.989354584 | 0.978822492 | 0.807949477 | 0.652782358 |
| Cerebelum_7b_L | 0.994813081 | 0.989653066 | 0.834472071 | 0.696343638 |
| Cerebelum_7b_R | 0.992899648 | 0.985849712 | 0.784215022 | 0.614993201 |
| Cerebelum_8_L | 0.995384625 | 0.990790551 | 0.880799404 | 0.775807591 |
| Cerebelum_8_R | 0.994509153 | 0.989048456 | 0.798774085 | 0.638040039 |
| Cerebelum_9_L | 0.988033855 | 0.976210899 | 0.862080848 | 0.743183388 |
| Cerebelum_9_R | 0.986711885 | 0.973600343 | 0.829163382 | 0.687511915 |
| Cerebelum_10_L | 0.983003735 | 0.966296343 | 0.755681967 | 0.571055235 |
| Cerebelum_10_R | 0.983175385 | 0.966633837 | 0.699435486 | 0.489209999 |
| Vermis_1_2 | 0.987216469 | 0.974596356 | 0.853925258 | 0.729188345 |
| Vermis_3 | 0.980156789 | 0.960707331 | 0.78685897 | 0.619147039 |
| Vermis_4_5 | 0.987346531 | 0.974853172 | 0.728016319 | 0.53000776 |
| Vermis_6 | 0.997644477 | 0.995294502 | 0.880166806 | 0.774693607 |
| Vermis_7 | 0.995131255 | 0.990286214 | 0.830461422 | 0.689666174 |
| Vermis_8 | 0.991980457 | 0.984025228 | 0.825040455 | 0.680691752 |
| Vermis_9 | 0.99571453 | 0.991447426 | 0.822752269 | 0.676921296 |
| Vermis_10 | 0.985006985 | 0.970238761 | 0.858117954 | 0.736366423 |
