# Supplementary material for: Modeling Brain Volume Using Deep Learning-Based Physical Activity Features in Patients With Dementia
Source: Front Neuroinform. 2022 Mar 9;16:795171. doi: 10.3389/fninf.2022.795171 (PMC8959707; doi:10.3389/fninf.2022.795171)
Supplement: Supplementary file 5 [file Image_1.PDF]

Supplementary Figure 1. Reconstructed data from the autoencoder model

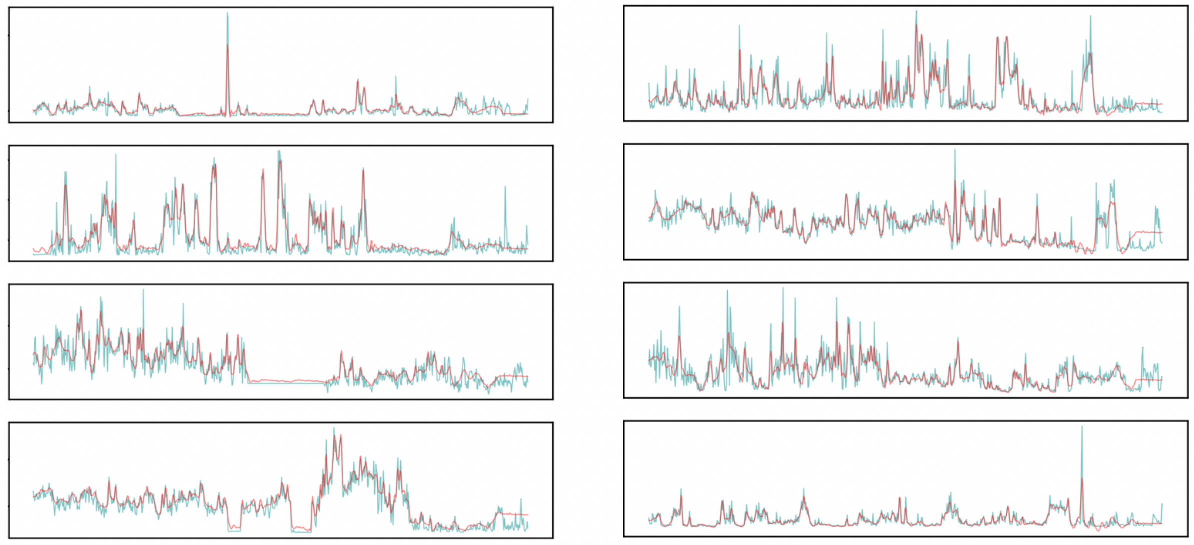

When we calculated the metrics of the autoencoder model, RMSE and MAE were 0.082 and 0.044 in the NHANES dataset (Training dataset), and 0.149 and 0.101 in the BICWALZS dataset (Test dataset). To validate autoencoder model in test dataset, we visualized the actual activity data from the accelerometer (cyan) and reconstructed activity data from the autoencoder (red) in BICWALZS dataset. In the eight independent sample sets shown above, x and y axes represent time and amount of activity, respectively.
